# Engineering resilient roses: molecular insights into biotic and abiotic stress adaptation

**DOI:** 10.1093/hr/uhaf332

**Published:** 2025-12-03

**Authors:** Hammad Hussain, Hamza Sohail, Edvinas Misiukevičius, Kaikai Zhu, Yazheng Cao, Yuqing Gu, Qianxiang Zhang, Yong Xu, Mengjuan Bai, Jianwen Wang, Guo Wei, Liguo Feng

**Affiliations:** College of Horticulture and Landscape Architecture, Yangzhou University, Yangzhou 225009, China; College of Horticulture and Landscape Architecture, Yangzhou University, Yangzhou 225009, China; Institute of Horticulture, Department of Orchard Plant Genetics and Biotechnology, Lithuanian Research Centre for Agriculture and Forestry, Kaunas District, LT-54333 Babtai, Lithuania; National Key Laboratory for the Development and Utilization of Forest Food Resources, Nanjing Forestry University, Nanjing 210037, China; College of Horticulture and Landscape Architecture, Yangzhou University, Yangzhou 225009, China; College of Horticulture and Landscape Architecture, Yangzhou University, Yangzhou 225009, China; College of Horticulture and Landscape Architecture, Yangzhou University, Yangzhou 225009, China; College of Horticulture and Landscape Architecture, Yangzhou University, Yangzhou 225009, China; College of Horticulture and Landscape Architecture, Yangzhou University, Yangzhou 225009, China; College of Horticulture and Landscape Architecture, Yangzhou University, Yangzhou 225009, China; College of Horticulture and Landscape Architecture, Yangzhou University, Yangzhou 225009, China; College of Horticulture and Landscape Architecture, Yangzhou University, Yangzhou 225009, China

## Abstract

Rose (*Rosa* spp.) is a high-value ornamental plant cultivated worldwide for its aesthetic and commercial importance. However, rose production is frequently challenged by a wide range of biotic and abiotic stresses that impair growth, development, and floral quality, ultimately reducing the yield and economic returns. Recent advances have clarified the molecular pathways that govern stress responses in roses, with particular emphasis on transcriptional regulation, post-translational protein modifications, and epigenetic control. Transcription factors such as the WRKY, NAC, MYB, and AP2/ERF families regulate stress-responsive gene expression. Post-translational modifications, including phosphorylation and ubiquitination, together with epigenetic mechanisms such as DNA methylation and chromatin remodeling, establish molecular ‘stress memory’ and resilience. In response to biotic stress, roses defend against major pathogens, including black spot (*Marssonina rosae*), gray mold (*Botrytis cinerea*), and powdery mildew (*Podosphaera pannosa*) through integrated hormonal signaling and transcriptional regulation. Aphid herbivory triggers calcium fluxes, phosphorylation cascades, and the synthesis of secondary metabolites that strengthen defense. Emerging biotechnological tools, particularly genome editing using clustered regularly interspaced short palindromic repeats/Clustered Regularly Interspaced Short Palindromic Repeats/CRISPR-associated protein 9, marker-assisted selection, and virus-induced gene silencing, provide promising approaches for breeding rose cultivars with improved tolerance to environmental and pathogenic stresses. This review synthesizes recent advances in understanding the molecular mechanisms underlying both biotic and abiotic stress adaptation in roses and outlines strategies for developing resilient cultivars capable of maintaining productivity and ornamental value under adverse conditions.

## Introduction

Rose (*Rosa* spp.), famously known as the ‘King of Flowers,’ ranks among the most admired ornamental species in the *Rosaceae* family. It is widely esteemed for its aesthetic appeal and economic importance, with the global rose market valued at approximately USD 28 billion [1]. Roses are cultivated and traded worldwide in various forms, including cut flowers, potted plants, and landscape ornamentals. Historical records trace rose cultivation to around 3000 BCE in regions such as China, North Africa, and Western Asia. However, *Rosa* spp. from China were only introduced into Europe during the 14^th^ century. The genus *Rosa* L. displays remarkable diversity, comprising more than 200 species and approximately 30 000–35 000 cultivars [2]. Initially, wild roses served functional purposes such as natural fencing for livestock, but their value soon extended to ornamental and medicinal applications across Roman, Greek, and Persian civilizations [3]. Beyond their aesthetic value, roses have become integral to high-value industries, including perfumery, pharmaceuticals, and cosmetics, further elevating their economic relevance [4–6]. They are rich sources of health-promoting phytochemicals such as vitamins, flavonoids, polyphenols, and polysaccharides. These bioactive constituents have been associated with the prevention and mitigation of chronic degenerative diseases, including cancer, diabetes, and cognitive decline [7]. Recently, rose petals have also gained attention in the food industry as functional ingredients in beverages, soups, and salads due to their distinctive flavor and aromatic properties [8].

Plants are constantly exposed to a range of biotic and abiotic stressors, many of which have intensified because of environmental pollution and accelerating climate change. These stressors pose serious risks to ecosystem stability and biodiversity [9]. The increasing frequency and severity of stress events under current climatic conditions have significantly constrained global crop productivity by disrupting normal physiological functions [10]. In their natural habitats, roses face numerous biotic challenges, including insect pests such as aphids and thrips and fungal pathogens such as gray mold, black spot, and powdery mildew [11]. To counter these threats, roses employ both constitutive and inducible defense mechanisms. Constitutive defenses include structural barriers such as thorns, trichomes, and waxy cuticles. Inducible responses are activated upon attack, and these include hormonal signaling pathways mediated by jasmonic acid (JA) and salicylic acid (SA), together with the synthesis of secondary metabolites and volatile organic compounds (VOCs) that deter herbivores or attract their natural enemies [12–14]. These multilayered defenses systems reflect the complex dynamics of rose pathogen and rose insect interactions and emphasize the importance of integrating a biotic perspective when evaluating stress adaptation strategies.

Among abiotic constraints, salinity, drought, cold, and temperature extremes represent major environmental limitations to plant performance. Such stresses profoundly affect plant metabolic integrity by impairing fundamental physiological and molecular processes. Photosynthesis, nutrient uptake, hormonal signaling, and cellular homeostasis are disrupted, ultimately compromising growth, productivity, and resilience [15].

**Figure 1:**
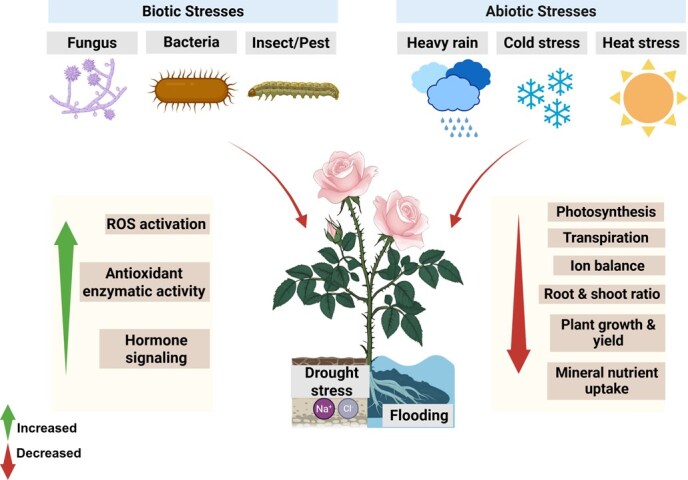
Representation of biotic and abiotic stress effects and adaptive responses in rose (*Rosa* spp.) plants. Various environmental and biological stresses significantly affect the growth and development of roses. These stressors trigger multiple physiological responses: increased ROS (Reactive Oxygen Species) activation, enhanced antioxidant enzymatic activity, altered hormone signaling pathways, changes in photosynthetic efficiency, modified transpiration rates, disrupted ion balance, altered root-to-shoot ratio, decreased plant growth and yield, and impaired mineral nutrient uptake.

Climate change increasingly threatens the global rose industry. Shifting environmental conditions reduce the ornamental quality of roses and endanger the livelihoods of millions who depend on their cultivation and trade [16]. Roses are highly susceptible to both biotic and abiotic stresses (Fig. 1), including drought, salinity, cold, and heat, as well as biotic pressures such as aphids, thrips, gray mold (*Botrytis cinerea*), black spot (*Marssonina rosae*), and powdery mildew (*Podosphaera pannosa*) [11]. These stresses adversely affect plant growth and development, ultimately reducing marketable yield and economic value [17].

**Figure 2:**
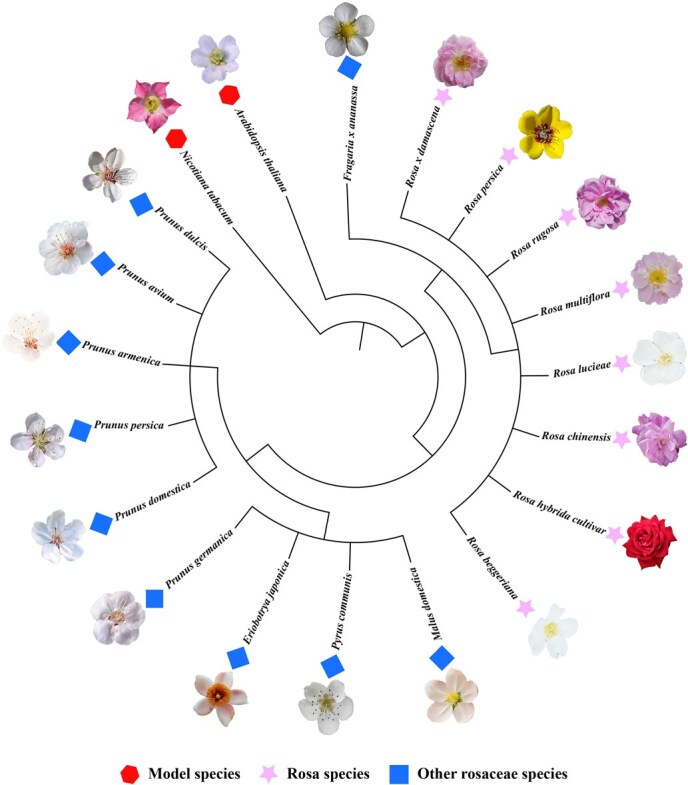
Phylogenetic relationships of selected *Rosa* species and related *Rosaceae* members. The topology was constructed to illustrate the evolutionary position of *Rosa chinensis* (*R. chinensis*), *Rosa multiflora* (*R. multiflora*), *Rosa rugosa* (*R. rugosa*), *Rosa damascena* (*R. damascena*), *Rosa persica* (*R. persica*), *Rosa lucieae* (*R. lucieae*), *Rosa beggeriana* (*R. beggeriana*), and *Rosa hybrida* (*R. hybrida*) cultivars in comparison with other *Rosaceae* species and model plants. Background symbols represent different groups: red hexagons = model species, pink stars = *Rosa* species, and blue diamonds = other *Rosaceae* species. This phylogenetic framework highlights the comparative evolutionary context for understanding stress response mechanisms in roses. Floral images of *Rosa* species were reproduced with permission from Cheng *et al.* [205]; images of the model and other *Rosaceae* members were obtained from publicly available repositories.

In the global marketplace, intense competition drives efforts to produce high-quality rose cultivars. Rose quality is evaluated through several parameters, including visual characteristics, postharvest performance, adaptability to various environmental conditions, and resistance to biotic and abiotic factors [18, 19]. These demands highlight the urgent need to elucidate both the physiological and molecular bases of rose responses to stress, enabling sustainable cultivation and the development of resilient varieties. Two principal strategies are currently utilized to mitigate stress in ornamental and floriculture crop systems: refining agronomic practices and generating stress-tolerant cultivars. However, traditional rose breeding is often constrained by intrinsic barriers such as self-incompatibility and prolonged juvenile phases, rendering conventional approaches labor-intensive and time-consuming. Modern biotechnological tools offer viable alternatives for enhancing stress tolerance in ornamental crops [18]. Understanding the molecular mechanisms underlying rose responses to biotic and abiotic stress is therefore crucial for targeted breeding programs aimed at improving resilience and ensuring stable yield and quality. Recent studies have examined how roses perceive environmental and biological stress signals and relay these cues to downstream effectors, triggering adaptive responses (Fig. 1). These investigations have revealed multilayered regulatory processes, including transcriptional reprogramming, post-translational modifications (PTMs), and epigenetic remodeling, which collectively modulate stress responses. To contextualize the genetic and phylogenetic placement of *Rosa* spp. Within the *Rosaceae* family and relative to model species, a comparative phylogenetic framework is presented (Fig. 2), establishing the evolutionary foundation for subsequent discussion of stress adaptation and breeding. This review summarizes recent progress in clarifying molecular regulatory networks governing rose adaptation to biotic and abiotic stress. It also highlights promising genetic and epigenetic targets for developing stress-resilient rose cultivars to enhance production efficiency and floral quality.

### Transcriptomic and physiological insights into abiotic stress responses in rose plants

Recent advances in transcriptomic and physiological research have substantially expanded understanding of the molecular and adaptive responses governing abiotic stress tolerance in roses. These studies provide a foundation for enhancing the resilience of rose cultivars against environmental challenges such as drought, salinity, cold, and heat. Drought stress is among the most extensively investigated abiotic factors in roses. Li *et al*. performed full-length transcriptome sequencing in *R. chinensis* under drought conditions, revealing marked changes in photosynthetic performance and phytohormone distribution. Under mild drought, both chlorophyll content and water use efficiency increased. Abscisic acid (ABA) accumulated primarily in leaf tissues, whereas auxin-related compounds such as indole-3-acetic acid (IAA), methylindole-3-acetic acid, and indole-3-propionic acid were enriched in the roots [20]. Similarly, Zhao *et al*. reported that drought stress in *R. hybrida* ‘Rouge Meilland’ reduced leaf expansion, root growth, and chlorophyll, while inducing excessive accumulation of reactive oxygen species (ROS) and suppressing antioxidant enzyme activities. To counter osmotic stress, roses activate the synthesis of compatible solutes [21]. Adamipour *et al*. reported that *R. canina* accumulated markedly higher levels of proline under drought conditions than *R. damascena*, a response linked to the upregulation of *P5CR* and *P5CS*, key enzymes in proline biosynthesis and osmotic adjustment [22]. At the regulatory level, Jia *et al*. applied weighted gene co-expression network analysis (WGCNA) to identify drought-responsive molecular hubs. Their results showed coordinated activity among several phytohormonal pathways, including ABA, auxin, brassinosteroids (BR), cytokinin, ethylene (ET), JA, and SA, together with transcription factor (TF) families such as *WRKYs*, *MYBs*, *AP2/ERFs*, *ARFs*, *ACs*, and *bHLHs*. These regulators collectively orchestrate gene expression changes that are central to metabolic adjustment and stress adaptation [23].

Under saline conditions, Li *et al*. demonstrated that *R. chinensis* exposed to salt stress exhibited reduced dry matter accumulation, shorter roots, and fewer flowers. Nevertheless, the plants adopted an ion exclusion mechanism, sequestering sodium (Na^+^) and chloride (Cl^−^) ions in roots and stems to prevent leaf ion toxicity [24]. Cold stress responses have also been extensively characterized. Zhang *et al*. conducted de novo transcriptome assembly in *R. multiflora*, identifying numerous genes upregulated in response to low temperature, particularly those related to metabolism, transport, signal transduction, and transcriptional regulation [25]. These molecular insights provide a foundation for understanding abiotic stress adaptation mechanisms in roses. These molecular insights provide a foundation for understanding abiotic stress adaptation mechanisms in roses.

Secondary metabolites, particularly phenolic compounds and proanthocyanidins (PAs), are key contributors to stress tolerance in roses. Li *et al*. functionally characterized *RcMYBPA2*, a TF from *R. chinensis* that regulates PA biosynthesis. Overexpression of *RcMYBPA2* in tobacco resulted in increased PA accumulation and improved oxidative stress tolerance by enhancing ROS scavenging [26]. Likewise, Luo *et al*. found that *RrANR*, isolated from *R. rugosa*, promoted the accumulation of both PAs and ABA, contributing to enhanced stress resistance [27]. In *R. damascena*, drought stress significantly altered phenolic composition and increased the activity of antioxidant enzymes such as lipoxygenase (LOX) and acetylcholinesterase (AChE) [28]. Translation-related genes also play roles in abiotic stress responses. Jiang *et al*. identified an *eIF5A* gene from *R. chinensis* that exhibited high expression in leaves and conferred thermotolerance [29]. Xu *et al*. further showed that overexpression of *RceIF5A* in *Arabidopsis* enhanced tolerance to heat, osmotic, and oxidative stress through increased proline accumulation and antioxidant activity, promoting root development under stress conditions [30].

Collectively, these transcriptomic and physiological findings demonstrate the multifaceted nature of abiotic stress responses in roses. Integrating hormone signaling, transcriptional regulations, osmolyte metabolism, and secondary metabolite biosynthesis provides a robust framework for breeding and biotechnological strategies aimed at enhancing abiotic stress resilience in rose cultivars.

## Molecular insights under biotic stress of roses

In addition to abiotic stress factors, roses are also susceptible to a wide range of biotic stresses caused by pathogens and insects. These stresses pose significant challenges to rose cultivation, affecting development, growth, health, and lifespan [19]. The most common infectious diseases of roses include black spot (*Diplocarpon rosae*), gray mold (*B. cinerea*), powdery mildew (*P. pannosa*), downy mildew (*Peronospora sparsa*), spot anthracnose (*Sphaceloma rosarum*), crown gall (*Agrobacterium tumefaciens*), and rose rosette virus (*Emaravirus* sp.). Among insect pests, aphids and thrips are particularly damaging, especially in the production of cut and potted roses. At present, only a limited number of commercial cultivars exhibit substantial disease resistance [31], highlighting the urgent need to elucidate biotic stress tolerance mechanisms through omics-based approaches that can inform genetic engineering strategies for enhanced resistance.

Black spot disease, primarily caused by *Marssonina rosae* (*M. rosae*) and *Alternaria alternata* (*A. alternata*), represents one of the most severe threats to rose cultivation, compromising both growth and ornamental quality. Molecular studies reveal complex hormonal regulation during infection. In susceptible roses, SA levels rise during early *M. rosae* infection, followed by suppression of SA-dependent defense signaling that downregulates key pathogen interaction genes (*OPR3, ICS, NPR1, MYC2,* and *WRKY70*), facilitating *A. alternata* invasion. At later infection stages, the decline in SA-related gene expression attenuates SA-JA antagonism, enhancing *M. rosae* pathogenicity and sporulation [32]. Comparative transcriptomic and metabolomic analyses of susceptible (‘Antique Romantica’) and resistant (‘Princess Michael of Kent’) *R. hybrida* cultivars reveal distinct defense strategies. Susceptible genotypes exhibit strong immune activation through upregulation of pathogenesis-related (PR) genes and WRKY/AP2-ERF TFs, as well as enhanced cell wall remodeling and ABA signaling. Conversely, resistant cultivars rely on early-stage defense responses mediated by Golgi vesicle trafficking and peptide secretion, enabling efficient delivery of defense compounds to infection sites [33]. These mechanisms parallel findings in model plants. In rice, BR signaling exhibits bidirectional regulation, enhancing resistance to *Magnaporthe grisea* and *Xanthomonas oryzae* pv. *oryzae* [34], but reducing resistance to *Pythium graminicola* and rice black-streaked dwarf virus [35].

**Figure 3:**
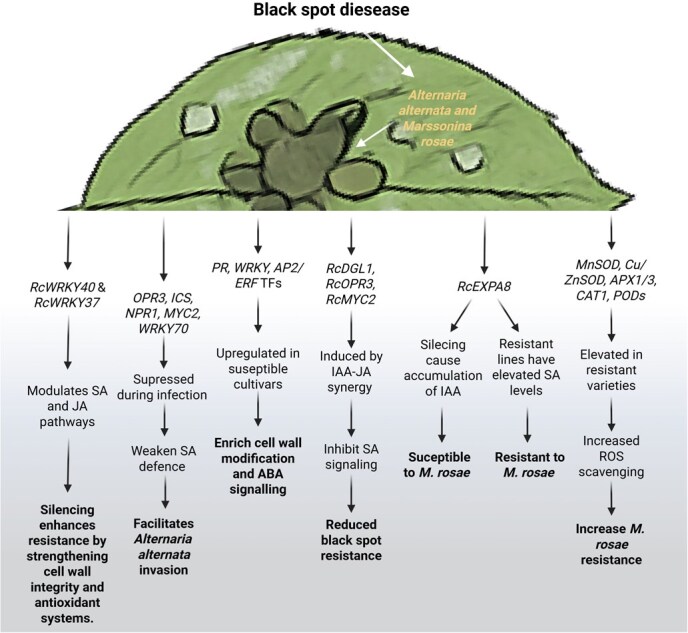
Molecular basis of black spot disease resistance and susceptibility in rose plants. Black spot disease caused by *A. alternata* and *M. rosae* triggers multiple molecular pathways that determine plant resistance or susceptibility. The figure shows how various TFs and genes respond to pathogen infection through distinct signaling cascades. Key resistance mechanisms include WRKY TFs (*RcWRKY40, RcWRKY37*) that modulate SA and JA pathways, leading to enhanced cell wall integrity and antioxidant defenses when silenced. Susceptibility pathways involve suppression of defense genes (*OPR3, ICS, NPR1*) and upregulation of stress-responsive TFs in vulnerable cultivars. The IAA-JA synergy pathway through *RoDGL1* and related genes inhibits SA signaling, reducing black spot resistance. *RcEXPA8* plays a dual role in both susceptibility and resistance mechanisms. Antioxidant enzymes (*MnSOD, Cu/ZnSOD, APX, CAT,* and *PODs*) are elevated in resistant varieties, enhancing ROS scavenging capacity. The integrated pathway demonstrates how molecular responses ultimately determine plant fate against black spot pathogens through complex regulatory networks governing defense signaling, cell wall modification, and oxidative stress management.

WRKY TFs emerge as central regulators of black spot resistance. *RcWRKY40* modulates resistance to *M. rosae* by balancing SA and JA pathways; its silencing enhances cell wall integrity and antioxidant defenses, thereby restricting pathogen invasion [36]. This mechanism is conserved in rice, where the *OsMKK4/OsMPK3/OsMPK6* cascade regulates *OsWRKY53* transactivation, and overexpression of phosphomimic *OsWRKY53* mutants induces major transcriptomic changes that activate defense responses against blast fungus [37]. In contrast, *RcWRKY37* functions as a pathogen-responsive factor without triggering SA-dependent pathways or PR gene expression [38], whereas *Arabidopsis AtWRKY28* and *AtWRKY75* primarily activate JA/ET pathways to defend against *Sclerotinia sclerotiorum* and oxalic acid stress [39]. Hormonal regulation of defense responses also reflects growth-immunity trade-offs. Antagonistic interactions between IAA and SA illustrate this balance: IAA promotes growth, whereas SA activates defense signaling [40]. Genome-wide analysis revealed that *RcEXPA8* silencing reduces *M. rosae* severity through hormonal modulation, resulting in elevated IAA levels in susceptible lines and increased SA accumulation in resistant ones [41]. A comparable mechanism occurs in rice, where activation of IAA-amido Synthetase GH3–8 suppresses expansin expression while promoting SA-JA-independent basal immunity [42]. Further complexity arises from JA-IAA synergism. Their co-accumulation inhibits SA signaling and decreases black spot resistance through IAA-induced expression of jasmonate biosynthesis genes *RcDGL1* and *RcOPR3*, accompanied by upregulation of *RcMYC2* [43]. Rose resistance mechanisms also depend on maintaining ROS homeostasis. Hydrogen peroxide (H₂O₂) accumulation is negatively correlated with disease resistance; resistant accessions exhibit higher antioxidant enzyme activities and increased expression of ROS-scavenging genes (*MnSOD, Cu/ZnSOD, APX1, APX3, CAT1, POD12, POD31, POD46, POD73*) during early *M. rosae* infection [44] (Fig. 3).

Gray mold, caused by the necrotrophic fungal pathogen *B. cinerea*, is among the most destructive diseases affecting roses, resulting in severe economic losses through necrosis, tissue collapse, and rot. Single-cell RNA sequencing of *R. hybrida* during *B. cinerea* infection reveals that epidermal tissues function as the primary physical defense barrier, whereas vascular tissues facilitate pathogen spread. PR protein RcFra and aquaporin *RcTIP2* act as positive regulators of resistance, whereas endochitinase RcEP3 functions as a negative regulator. WRKY TFs *RcWRKY22*, *RcWRKY24*, and *RcWRKY33* are significantly upregulated in bundle sheath cells via mitogen-activated protein kinase (MAPK) signaling, whereas pectin methylesterases, proline-rich proteins, and aquaporins (*TIP1–1, PIP2–1, PIP2–7*) are downregulated, weakening cell wall defenses and impairing water transport. Secondary metabolite biosynthesis, particularly Fra-mediated anthocyanin accumulation, provides temporary protection against necrotrophic infection. Genes associated with plant pathogen interactions are highly expressed in epidermal cells, especially those associated with cuticular wax biosynthesis such as *CUT1, KCS1,* and *KCS20*. Very-long-chain fatty acids (VLCFAs) serve as essential membrane components and surface barriers through 3-ketoacyl-CoA synthase (KCS)-mediated condensation of C(2) units into acyl-CoA [14]. These defense mechanisms parallel findings in model plants, where phosphorylation of *OsPIP2;2* regulates immunity via coordinated H₂O₂ transport and TF nuclear translocation [45]. In *Arabidopsis, KCS9* extends C22 to C24 fatty acids, providing precursors for cuticular wax, suberin, and membrane lipid biosynthesis, whereas *KCS3* acts as a negative regulator by reducing *KCS6* enzyme activity [46].

**Figure 4:**
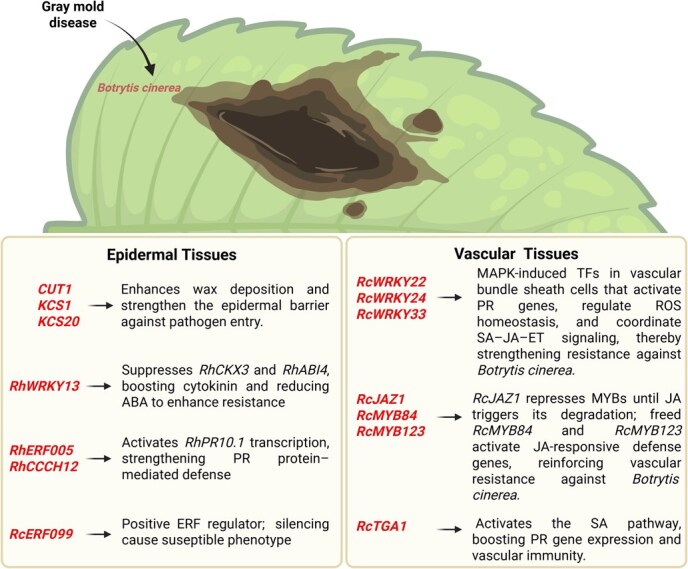
Rose immunity against gray mold disease is regulated by a complex network of genes. Gray mold disease caused by *B. cinerea* elicits distinct molecular responses in epidermal and vascular tissues. In epidermal tissues, multiple genes coordinate barrier defenses: *CUT1*, *KCS1*, and *KCS20* enhance wax deposition to strengthen the epidermal barrier against pathogen entry, while *RhWRKY13* suppresses cytokinin signaling genes (*RhCKX3, RhABI4*) and reduces ABA levels to enhance resistance. *RhERF005* and *RhCCCH12* activate PR protein *RhPR10.1* transcription, strengthening PR protein-mediated defense. *RcERF099* acts as a positive ERF regulator, with silencing leading to susceptible phenotypes. In vascular tissues, WRKY TFs (*RcWRKY22, RcWRKY24,* and *RcWRKY33*) function as MAPK-induced regulators in bundle sheath cells, activating PR genes and coordinating SA-JA-ET signaling pathways. The JA pathway involves *RcJAZ1* repressing MYB TFs until JA triggers degradation, releasing *RcMYB84* and *RcMYB123* to activate JA-responsive defense genes. *RcTGA1* activates SA pathway components, boosting PR gene expression and vascular immunity. This integrated system demonstrates how tissue-specific regulatory networks collectively determine plant resistance to *B. cinerea* through coordinated epidermal barrier reinforcement and vascular defense signaling.

The molecular architecture of gray mold resistance integrates multiple TF families. *AtWRKY33* functions as a pivotal regulator of *B. cinerea* resistance by targeting redox homeostasis, SA signaling, ET-JA cross-talk, and glucosinolate biosynthesis [47]. MAPK pathway activation regulates *AtWRKY33*-mediated phytoalexin biosynthesis to strengthen pathogen defense [48]. In *R. hybrida*, *RhWRKY13* binds to promoter regions of cytokinin degradation gene *CKX3* (*RhCKX3*) and ABA-response gene (*RhABI4*), repressing their expression in petals and thereby increasing cytokinin levels and reducing ABA signaling, which collectively enhance protection from *B. cinerea* [49]. This mechanism aligns with *Arabidopsis*, where the abi4–1 mutant with inactivated ABA signaling exhibits lower susceptibility to *B. cinerea* than wild-type plants [50]. Complementary to WRKY-mediated regulation, AP2/ERF TFs also contribute significantly to gray mold defense. Genome-wide analysis shows that *RcERF* genes are upregulated upon *B. cinerea* infection, and *RcERF099* acts as a positive regulator whose silencing increases disease susceptibility [51], consistent with ERF-mediated defense mechanisms reported in *Arabidopsis* and rice [52]. Additionally, ET-JA regulated TFs *RhEFR005* and *RhCCCH12* bind to the *RhPR10.1* promoter, promoting its transcription and reducing *B. cinerea* susceptibility [53]. This parallels *Arabidopsis* C3H14, a CCCH protein that enhances defense against *B. cinerea* by activating *WRKY33*-dependent signaling [54]. JA signaling further orchestrates defense through protein–protein interactions, where *RcJAZ1, RcMYB84,* and *RcMYB123* physically interact; JA treatment induces *RcJAZ1* degradation, releasing *RcMYB84* and *RcMYB123* to activate downstream defense responses [55]. Transcriptomic and metabolomic analysis of *R. chinensis* ‘Old Bush’ infected by *B. cinerea* revealed coordinated defense responses mediated by PR MAPK cascades, Ca^2+^ signaling, and plant pathogen interaction pathways. *RcTGA1* enhanced resistance through activation of the SA pathway, whereas secondary metabolites such as tannins, amino acids, and alkaloids accumulated through enriched phenylpropanoid and glucosinolate pathways [56]. This pattern is consistent with *Arabidopsis*, where *NPR1* interacts with the TGACG motif-binding transcription factor (TGA) to activate SA responsive elements in the promoter of *PR1* [57] (Fig. 4).

Powdery mildew, caused by *P. pannosa*, is another devastating fungal disease that primarily affects young rose leaves and leads to serious economic losses. Molecular analyses indicate that exogenous SA application reduces *P. pannosa* infection through pathogen-associated molecular pattern (PAMP) recognition, mediated by upregulated chitin recognition proteins, EIX1/2, and WAK. These processes activate pattern-triggered immunity (PTI) and effector-triggered immunity (ETI) through calcium MAPK signaling cascades. Defense responses feature enhanced secondary metabolite biosynthesis across sesquiterpenoid, flavonoid, glucosinolate, and phenylpropanoid pathways, where SA signaling interacts positively with MAPK and CaM-mediated resistance networks against biotrophic pathogens [58]. In *R. hybrida*, ET-mediated susceptibility to *P. pannosa* arises from pathogen-induced downregulation of ET biosynthesis genes, whereas treatment with the anti-ET 1-MCP enhances resistance by blocking ET perception [59]. In contrast, resistance in *R. multiflora* correlates with early activation of the SA pathway and increased chitinase expression, whereas susceptible genotypes rely on JA/ET signaling, with phenylpropanoid biosynthesis and callose deposition contributing to cell wall-based defense [60]. TCP TFs, *RcTCP2,* and *RcTCP9* act as positive regulators of powdery mildew resistance, showing upregulation in resistant varieties and downregulation in others, similar to *Arabidopsis TCP15*, which regulates immunity via *MOS1* interaction and *SNC1* promoter binding [61, 62]. Silencing *RhMLO*1 in *R. multiflora* through antisense transformation confirmed MLO as a susceptibility gene, enhancing powdery mildew resistance [63]. This mirrors *Arabidopsis thaliana*, where the *mlo2, mlo6,* and *mlo12* triple mutant exhibits complete penetration resistance via rapid defense gene activation, demonstrating that MLO proteins serve as susceptibility factors enabling pathogen-mediated defense suppression during fungal invasion [64].

Insect herbivory, particularly by aphids, poses additional challenges to rose cultivation through direct tissue damage and viral disease transmission, severely affecting ornamental quality and plant health [13]. In response to herbivore attack, roses employ antixenosis, antibiosis, and tolerance mechanisms coordinated through calcium fluxes, phosphorylation cascades, and JA signaling, which induce the synthesis of defensive metabolites [65]. Following aphid herbivory, *RcLOX12* upregulation promotes JA biosynthesis, consistent with *Arabidopsis* LOX functions, whereas WRKY TFs (*RlWRKY10, RlWRKY14*) mediate JA/SA-dependent resistance [66–68]. Under *M. rosivorum* stress, *R. longicuspis* activates MAPK cascades, hormone signal transduction, *RlMYB* and *RlERF* expression, and ROS generation, with enrichment of BR biosynthesis and secondary metabolite pathways, including terpenoids, tannins, phenolic acids, and glucosinolates through glutathione and glucosinolate metabolism [12]. These responses parallel *Arabidopsis*, where aphid feeding induces synthesis of 4-methoxyindole-3-methylthiogluconate, and its exogenous application enhances aphid resistance [69]. Proteomic comparisons between resistant (‘Stella’, ‘Alibaba’) and susceptible (‘Sun Star’, ‘Haetsal’) *R. hybrida* cultivars show that resistance correlates with elevated stress-responsive proteins, including PR proteins and antioxidant enzymes such as superoxide dismutase (SOD), ascorbate peroxidase (APX), and catalase. Resistant cultivars exhibit superior ROS detoxification capacity and maintain higher carbohydrate reserves that may serve as signaling molecules or energy sources for sustained defense. Exogenous SA treatment enhances secondary metabolite accumulation and shortens aphid lifespan [70], emphasizing the cross-talk among hormone pathways in regulating rose defense responses.

## Transcriptional regulation in abiotic stress responses of roses

**Figure 5:**
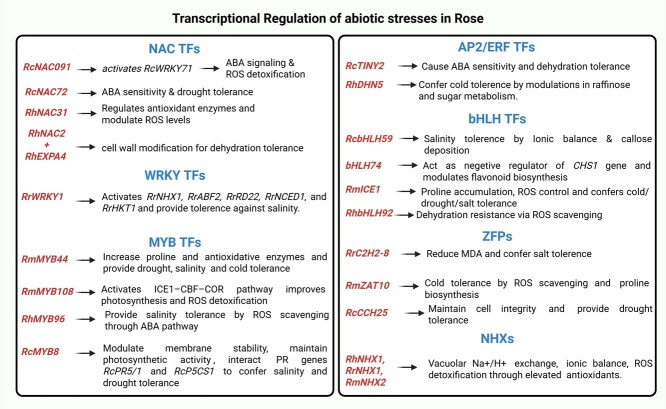
TF families regulating abiotic stress tolerance in rose plants. Multiple TF families coordinate rose responses to various abiotic stresses through distinct regulatory mechanisms. NAC TFs play central roles in stress adaptation: *RcNAC091* activates *RcWRKY71* to enhance ABA signaling and ROS detoxification, while *RcNAC72* mediates ABA sensitivity and drought tolerance. *RhNAC31* regulates antioxidant enzymes and ROS levels, and *RhNAC2/RhEXPA4* modifies cell walls for dehydration tolerance. WRKY TFs, particularly *RrWRKY1*, activate multiple stress-responsive genes (*RrNHX1, RrABF2, RrRD22, RrNCED1, RrHKT1*), providing salinity tolerance. MYB TFs regulate diverse stress responses: *RmMYB44* enhances proline and antioxidant production for multiple stress tolerances, *RmMYB108* activates the ICE1-CBF-COR pathway, improving photosynthesis and ROS detoxification, while *RhMYB96* and *RcMYB8* provide salinity tolerance through ROS scavenging and membrane stability maintenance. AP2/ERF factors (*RcTINY2, RhDHN5*) confer ABA sensitivity, dehydration, and cold tolerance through metabolic modulations. bHLH TFs regulate ionic balance and secondary metabolism, with RcbHLH59 providing salinity tolerance and *bHLH74* acting as a negative regulator of flavonoid biosynthesis. ZFPs and NHXs complete the regulatory network through ROS control, proline biosynthesis, and ionic homeostasis, demonstrating the integrated transcriptional control of abiotic stress responses in rose plants.

In recent years, transcriptomic approaches have become indispensable for elucidating plant responses to biotic and abiotic stresses [71]. These high-throughput analyses provide comprehensive insights into the regulatory networks and gene expression dynamics that underlie plant stress adaptation, including in roses. A major outcome of transcriptome profiling is the identification of TFs, which act as key regulators of stress-responsive gene expression [72]. In roses, transcriptional regulation functions through a finely coordinated network of TFs and downstream stress-responsive genes (Fig. 5). These TFs act as molecular switches, binding to cis-regulatory elements within promoter regions to activate or repress transcription in response to environmental stimuli [73]. Structurally, TFs contain conserved DNA-binding domains that recognize specific promoter motifs, nuclear localization signals for intracellular targeting, and regulatory domains that determine stress-specific activity [74].

Multiple TF families have been implicated in abiotic stress tolerance in *Rosa* spp., including WRKY proteins characterized by the conserved WRKYGQK motif [75, 76]. NAC family members such as NAM, ATAF1/2, and CUC2 [77–79]. MYB domain-containing proteins [80, 81] and heat shock factors (HSFs) [82–84]. Additional TF families include APETALA2/ethylene responsive factors (AP2/ERFs) [85–87], zinc finger proteins (ZFPs) [88, 89], DNA-binding with one finger (Dof) proteins [90], basic helix–loop–helix (bHLH) factors [61, 91–93], lateral organ boundaries domain (LBD) proteins [94], and basic leucine zipper (bZIP) factors. Moreover, the sodium/hydrogen exchanger (NHX) gene family, though not classical TFs, contributes significantly to abiotic stress responses by maintaining ion homeostasis [95, 96]. Collectively, members of these TF families exhibit rapid and dynamic expression changes under environmental stress, highlighting their central roles in rose stress physiology.

## NAC regulons

The NAC (NAM, ATAF1/2, and CUC2) TF family is one of the largest plant-specific TF families and plays diverse roles in plant development and abiotic stress adaptation. Based on sequence characteristics, NACs are classified into 18 subgroups, including well-studied subfamilies such as NAP, NAM/CUC3, SNAC, and VNS, each with distinct functions in organ development and stress signaling [79]. NAC TFs regulate processes such as root and shoot development, floral morphogenesis, senescence, fruit ripening, and responses to drought, salinity, and cold stress [97]. In roses, genome-wide characterization has identified several NAC genes associated with abiotic stress tolerance. Geng *et al*. identified *RcNAC091* in *R. chinensis* as a major regulator of drought and salt stress. Silencing *RcNAC091* reduced dehydration tolerance in leaves, resulting in greater ion leakage and chlorophyll degradation [78]. Further analysis revealed that *RcNAC091* directly regulates *RcWRKY71*, activating stress and ABA-related downstream genes that enhance ROS detoxification and promote stomatal closure [79]. Similarly, *RcNAC72* from *R. chinensis* ‘Old Blush’ was reported to confer drought tolerance and ABA sensitivity; overexpression in *A. thaliana* improved water retention and survival under drought, whereas silencing impaired leaf expansion and dehydration response [98]. Recent findings by Fu *et al.* demonstrated that *RhNAC31* enhances drought tolerance in *R. hybrida* by regulating stress-related genes. *RhNIRF1,* a RING-type E3 ligase, targets *RhNAC31* for degradation, thereby modulating its transcriptional activity during drought. Overexpression of *RhNAC31* improved photosynthetic performance and reduced oxidative stress, confirming its potential for enhancing drought resilience in roses [99].

Additional NAC genes have been functionally characterized in *R. hybrida*. Ding *et al*. identified *RhNAC31* as a regulator of flower opening and tolerance to dehydration, salt, and cold. Overexpression in *Arabidopsis* enhanced survival under stress by modulating ROS levels, malondialdehyde (MDA) content, and antioxidant enzyme activities, including POD and SOD [100]. Similarly, Jiang *et al*. showed that *RhNAC3 is* induced by dehydration, ABA, ET, and wounding. Silencing *RhNAC3* in rose petals reduced dehydration resistance, whereas its overexpression in *Arabidopsis* upregulated osmotic stress-related genes [77]. Petal expansion represents another stress-sensitive trait in cut roses. Dai *et al*. identified *RhNAC2* and *RhEXPA4* in the petals of *R. hybrida* cv. ‘Samantha,’ revealing their roles in dehydration resistance through regulation of cell wall-related genes [101]. Overexpression of *RhEXPA4* in *Arabidopsis* conferred salt and drought tolerance by modifying leaf anatomy, increasing lateral root formation, and enhancing chlorophyll A content [102]. Collectively, these studies demonstrate the integral role of NAC TFs in orchestrating complex, multifaceted stress responses in rose species.

## NHX regulons

Soil salinization represents a major limitation to agricultural productivity, affecting nearly 20% of irrigated land worldwide. Under saline conditions, plants experience cellular ion imbalance, oxidative damage, and impaired water uptake, which collectively restrict growth and development [103]. To counter these effects, plants have evolved multiple adaptive mechanisms, including ion sequestration, compartmentalization, exclusion, and hormonal regulation [104]. Among these, sodium/hydrogen exchangers (NHXs) play a pivotal role. These antiporters belong to the cation/proton antiporter 1 (CPA1) superfamily and trace their evolutionary origin to NhaP genes in prokaryotes [105]. The first plant NHX gene was cloned from *A. thaliana* (*AtNHX1*), marking a major advance in understanding plant ion homeostasis [106]. In eukaryotic systems, NHX proteins are localized primarily to the tonoplast membrane, where they mediate Na^+^/H^+^ exchange to maintain pH and ionic balance within vacuoles. This sequestration of Na^+^ protects cytosolic enzymes from toxicity and preserves cellular turgor under salinity stress [107]. NHX-mediated salinity tolerance also depends on K^+^ retention and vacuolar pH regulation, offsetting which counteracts acidification caused by proton pumps [108]. Sodium influx into plant cells occurs mainly through voltage-dependent and voltage-independent non-selective cation channels (NSCCs), requiring rapid and efficient detoxification mechanisms to maintain cellular homeostasis.

In ornamental roses, several NHX homologs have been characterized. *RhNHX1*, identified in *R. hybrida*, encodes a vacuolar Na^+^/H^+^ antiporter capable of complementing salt-sensitive yeast nhx1 mutants [109]. Its expression is markedly upregulated under NaCl stress, indicating a direct role in salinity tolerance. In *R. rugosa*, *RrNHX1* and the vacuolar H^+^-ATPase subunit *RrVHA-c* jointly enhance salt tolerance [95]. These findings were later supported by Reis *et al*. who confirmed the role of *RrNHX1* and its association with the expansin gene *EXP4* in mitigating salt-induced damage [85]. A study by Luo *et al*. in *R. multiflora*, among which *RmNHX2* exhibited the highest salt-responsive expression. Functional validation demonstrated that *RmNHX2* overexpression in tobacco improved ion homeostasis, reduced ROS accumulation, and enhanced antioxidant enzyme activities [96]. In contrast, silencing of *RmNHX2* increased salt sensitivity, confirming its positive regulatory function in salinity tolerance.

## *WRKY* regulons

The WRKY TF family plays a central role in regulating plant responses to diverse abiotic stresses. First identified in *Ipomoea batatas* through the *SPF1* gene, WRKY TFs have since been characterized across many plant species, including ornamentals [110, 111]. WRKY proteins contain a conserved WRKY domain (~60–70 amino acids) featuring the WRKYGQK heptapeptide and a zinc-finger motif, typically of the C_2_H_2_ or C2HC type. Based on the number of WRKY domains and zinc-finger configurations, WRKY proteins are grouped into three major classes: Group I (two WRKY domains), Group II (one WRKY domain and C_2_H_2_-type zinc finger, subdivided into IIa–IIe), and Group III (one WRKY domain with a C2HC-type zinc finger) [112]. Functionally, WRKY TFs regulate plant adaptation to drought, salinity, and low temperature by modulating the expression of defense and stress-related genes. They are also integrated into hormonal signaling pathways, particularly those mediated by ABA and gibberellic acid (GA), thereby influencing growth and stress responses [113].

Recent functional genomics research has clarified the roles of WRKY TFs in rose abiotic stress adaptation. In *R. chinensis*, genome-wide identification revealed that *RcWRKY27* and *RcWRKY29* participate in alkaline stress tolerance and developmental regulation [75]. Notably, *RcWRKY49* expression is strongly induced under salt stress, suggesting its involvement in ion homeostasis [76]. In *R. rugosa*, the salt-inducible gene *RrWRKY1* is expressed predominantly in leaves and petals. Silencing *RrWRKY1* caused increased leaf desiccation, vein blackening, and reduced enzymatic antioxidant activities (SOD, POD), accompanied by MDA content. Conversely, *RrWRKY1* overexpression in *A. thaliana* enhanced seed germination and lateral root formation, coinciding with upregulation of salt-responsive genes including *RrNHX1*, *RrABF2*, *RrRD22*, *RrNCED1*, and *RrHKT1*. These findings highlight the multifaceted regulatory role of *RrWRKY1* in salt stress adaptation [114].

## Zinc-finger protein regulons

Zinc-finger TFs constitute a major class of plant regulatory proteins characterized by zinc-coordinating domains that enable DNA and RNA binding. These proteins are classified according to the arrangement of histidine and cysteine residues into C_2_H_2_, C2HC, C3H, and CCCH types [115]. Among these, the C_2_H_2_-type is particularly abundant in plants and plays key roles in development, hormone signaling, and abiotic stress adaptation [116, 117]. Several zinc-finger proteins have been functionally characterized in rose species for their contribution to abiotic stress tolerance. In *R. rugosa*, the C_2_H_2_-type zinc-finger protein *RrC_2_H_2_–8* improved salt tolerance when overexpressed in *Arabidopsis*, markedly reducing MDA accumulation and cellular damage under salinity stress [89]. Similarly, *RmZAT10* was identified as a cold-responsive TF in *R. multiflora*. Functional validation through overexpression in tobacco and gene silencing in *R. multiflora* confirmed its role in enhancing cold tolerance by regulating proline biosynthesis and maintaining ROS homeostasis [118]. In *R. chinensis*, a genome-wide analysis identified 41 members of the CCHC-type zinc-finger family, categorized into five groups, with Group 4 genes showing pronounced upregulation under drought conditions in both leaves and roots. Silencing of *RcCCHC25* produced phenotypes such as leaf curling, brittleness, and increased ion leakage, indicating its role in preserving cellular integrity during water deficit [88]. Additionally, *RcC3H,* expressed predominantly in root and leaf tissues, was associated with root architecture, leaf senescence, and flowering regulation. Physiological assessments further indicated its involvement in salinity and hypoxia responses [117].

## Heat stress regulons

Rising global temperatures presents a growing challenge for plant productivity, with heat stress severely disrupting physiological and molecular processes. Plants counter these effects through the induction of heat shock proteins (HSPs), which act as molecular chaperones to stabilize proteins and membranes. HSPs are classified by molecular mass into five major families: small heat shock proteins (sHSPs), HSP60, HSP70, HSP90, and HSP100 [119]. Elevated temperatures increase ROS generation, impair photosynthetic performance, and destabilize cellular membranes [120]. To mitigate these effects, plants activate stress-responsive mechanisms that sustain redox balance and cellular homeostasis [121]. In *R. chinensis*, the cytosolic class I sHSP *RcHSP17.8* showed broad stress inducibility. Overexpression in *E. coli*, yeast, and *Arabidopsis* conferred tolerance to multiple abiotic stresses by enhancing SOD activity, reducing electrolyte leakage, and promoting root growth [122]. Comparative transcriptomic studies between *R. multiflora* (heat-tolerant) and *R. chinensis* (heat-sensitive) revealed activation of HSF and HSP gene families under high temperature, accompanied by increased osmolyte accumulation and SOD activity [71]. Further transcriptomic analysis of *R. chinensis* showed rapid upregulation of genes related to protein folding, ROS detoxification, and compatible solute biosynthesis, whereas genes associated with photosynthesis and cell wall metabolism were downregulated [123]. Genome-wide analyses identified key HSP family members, including *RcHSP90–1-1*, *RcHSP90–5-1*, and *RcHSP90–6-1*, that contribute to salinity and drought stress regulation [84]. Similarly, overexpression of *RcHSP70* in *R. hybrida* enhanced both thermotolerance and cold resistance in tobacco [82]. A recent study by Sun *et al.* identified *RhHsfA7* as a major regulator of heat adaptation in hybrid roses. Overexpression of *RhHsfA7* significantly improved thermotolerance, whereas silencing reduced heat resilience, highlighting the key function of HSFs in controlling thermal stress responses [124]. Integrated omics studies further demonstrated that thermotolerance in roses depends on coordinated regulation among ET signaling, flavonoid biosynthesis, and MAPK pathways, with *RcHSP70* acting as a central component of the heat-response network [83].

## *MYB* regulons

The MYB TF family represents one of the largest and most diverse groups in plants, comprising regulators that control growth, development, secondary metabolism, and stress responses. Structurally, MYB TFs are defined by one to four imperfect MYB repeats (R1-R4), each about 52 amino acids in length, forming a helix-turn-helix configuration that facilitates DNA binding [125]. Based on repeat number, MYB TFs are categorized into four main subgroups: 1R-MYB (MYB-related), R2R3-MYB, 3R-MYB, and 4R-MYB. The R2R3-MYB subclass is the most prevalent in higher plants and is frequently associated with abiotic stress adaptation. MYB TFs mediate plant tolerance to drought, salinity, and cold by regulating genes associated with secondary metabolism, antioxidant defense, hormone signaling, and osmotic adjustment [126, 127]. In *R. multiflora*, *RmMYB44* expression was upregulated under drought, salinity, and cold treatments. Overexpression in tobacco conferred enhanced stress tolerance through increased proline content and antioxidant enzyme activities (SOD, POD, and catalase [CAT] and decreased MDA and hydrogen peroxide levels) [81]. Geng *et al.* demonstrated that the JA signaling pathway and ICE-CBF-COR cascade are integral to cold tolerance in *R. persica*. Their findings identified *RpMYC2* as a key TF interacting with multiple regulators to modulate cold response [128]. Similarly, *RmMYB108*, expressed mainly in roots, enhanced cold tolerance in *Arabidopsis* by activating ICE1, CBF, and COR genes, thereby improving photosynthetic efficiency and ROS detoxification [129].

In *R. hybrida*, *RhMYB96* functions in a stage-specific manner to confer salt tolerance. Its overexpression in *Arabidopsis* improved germination, root development, and ROS scavenging via ABA-mediated signaling [130]. Another MYB TF, *RcMYB8* from *R. chinensis*, regulates drought and salt tolerance by maintaining membrane stability and photosynthetic activity while interacting with key stress-related proteins, including *RcPR5/1* and *RcP5CS1*. The latter being essential for proline biosynthesis and ROS control under dehydration stress [80]. Furthermore, in *R. chinensis* cv. ‘Old Blush,’ multiple MYB TFs (*MYB4*, *MYB14*, *MYB20*, *MYB41*, *MYB44*, and *MYB62*) were differentially expressed under salt stress, suggesting their roles in complex stress adaptation networks [131].

## APETALA2/ethylene responsive factor (*AP2/ERF*) regulons

The AP2/ERF TF family is one of the most extensive plant-specific regulatory groups, playing critical roles in development and environmental stress adaptation. These TFs act as key components of stress signal transduction pathways, rapidly activating or repressing downstream gene expression in response to abiotic cues [132]. Structurally, the AP2/ERF family is divided into four subfamilies: AP2, ERF, RAV, and DREB, with the latter two being particularly important for stress responses. DREB proteins, which are highly conserved among plant species, are further categorized into six subgroups (A-1 to A-6) based on sequence similarity and functions [133, 134]. In roses, AP2/ERF family members have been implicated in multiple abiotic stress responses. In *hybrid tea roses*, genome-wide screening identified *RhDREB36* and *RhERF59*, both showing sequence similarity to *Arabidopsis* stress-responsive genes *AtDREB2A* and *AtDREB2C*, suggesting a conserved role in drought tolerance [87]. In *R. chinensis*, *RcTINY2*, a DREB subfamily member, exhibited tissue-specific expression under stress: it was induced by ABA in leaves but suppressed in roots during ABA, drought, and salt treatments. Functional assays revealed that *RcTINY2* enhances ABA sensitivity during germination and regulates root growth under stress, whereas silencing reduced dehydration and salinity tolerance [86].

Another DREB member, *RcDREB2B*, was downregulated under both mild and severe drought in *R. chinensis*. Overexpression in *Arabidopsis* rendered seedlings hypersensitive to ABA and osmotic stress, resulting in lower expression of stress-responsive genes [133]. Comparative transcriptomic analysis in *R. hybrida* indicated stronger and faster AP2/ERF gene activation in leaves than in floral buds during stress exposure [135]. Functional validation through transgenic approaches has further demonstrated their roles in stress adaptation. For example, *MtDREB1C* from *Medicago truncatula*, driven by the *rd29A* promoter, conferred cold tolerance in *R. chinensis* by enhancing proline and soluble sugar accumulation, photosynthetic efficiency, and water retention [136, 137]. Similarly, *AtDREB2ACA* expression in *R. chinensis* improved salt tolerance but led to altered leaf morphology and decreased chlorophyll and starch content [138]. Beyond transcriptional regulation, downstream effector genes such as dehydrins play essential roles in cold acclimation. *RhDHN5*, which is strongly induced under cold in rose cultivars ‘Dagmar Hastrup’ and ‘Chandos Beauty,’ coordinates with starch and sugar metabolism genes (*RhBAM3*, *RhRS6*, *RhGK*, *RhSPS1*, *RhHXK1*, *RhFRK4*) to regulate raffinose and sucrose metabolism, thereby promoting cold hardiness [139].

## *bHLH* regulons

The bHLH family represents one of the largest and most functionally diverse TF groups in plants, regulating growth, development, and adaptation to abiotic stresses [140]. bHLH proteins possess two characteristic domains: a basic region at the N-terminus responsible for binding E-box (CANNTG) sequences in target gene promoters and an HLH domain at the C-terminus that mediates protein–protein interactions [141]. Functionally, bHLH TFs participate in cell elongation, stomatal regulation, hormone signaling, and secondary metabolism, and they play prominent roles in defense against salinity, drought, and cold stress [91, 142]. In *R. chinensis*, *RcbHLH59* enhances salt tolerance by modulating the *RcbHLH59–RcPRs* regulatory module, which maintains ion homeostasis and regulates callose deposition [92]. A multi-omics comparative study of *R. hybrida* cv. ‘Jardin de Granville’ (JDG) and *R. damascena* Mill. (DMS) revealed that salt tolerance in JDG is associated with activation of the phenylpropanoid pathway, particularly flavonoid biosynthesis. Within this pathway, *bHLHL74* functions as a negative regulator of the *CHS1* gene, modulating flavonoid accumulation under salt stress [6].

Another well-characterized member, *RmICE1* from *R. multiflora*, acts as a MYC-like regulator mediating cold, drought, and salt tolerance. Heterologous expression of *RmICE1* in tobacco conferred enhanced stress resilience, with lower MDA content, reduced electrolyte leakage, higher antioxidant enzyme activities, and increased proline levels [91]. In *R. hybrida*, *RhbHLH92* is specifically expressed in petals under dehydration stress; its overexpression improved water retention and antioxidant capacity, whereas silencing led to oxidative damage and loss of tissue turgor under drought conditions [143]. Furthermore, genome-wide analysis in *R. persica* identified *RbebHLH* genes that function in cold acclimation through jasmonate and ICE–CBF pathways influencing key physiological markers such as membrane conductivity and lipid peroxidation [144].

## Miscellaneous TFs families

TFs coordinate complex regulatory networks that enable plants to perceive and respond to environmental stimuli. Although extensive progress has been made in characterizing major TF families, several additional regulatory groups with key roles in abiotic stress tolerance remain less explored in *Rosa* species. The teosinte branched1/cycloidea/proliferating cell factor (TCP) family, unique to plants, participates in a broad range of physiological processes, including shoot branching, leaf morphogenesis, flowering, circadian rhythm regulation, and phytohormone signaling [145, 146]. Genome-wide analyses in *R. chinensis* identified five TCP genes (*RcTCP2*, *RcTCP4*, *RcTCP7*, *RcTCP14*, and *RcTCP15*) showing upregulated expression under heat, drought, and salinity stress [61].

Mediator proteins act as molecular bridges linking TFs to RNA polymerase II during transcription initiation. In *R. hybrida*, the mediator subunit *RhMED15a* enhanced osmotic stress tolerance when overexpressed in *Arabidopsis*, improving germination, root elongation, and biomass accumulation. Conversely, *RhMED15a* silencing increased oxidative damage and downregulated drought-responsive genes [147, 148]. Terpene synthases (TPSs) contribute to volatile compound biosynthesis, stress adaptation, and plant environment interactions [149]. Several *RcTPS* genes from *R. chinensis* displayed tissue-specific expression and differential regulation under osmotic and thermal stress. Notably, *RcTPS46* and *RcTPS01* were upregulated, whereas *RcTPS06*, *RcTPS16*, *RcTPS34*, *RcTPS36*, and *RcTPS44* were downregulated under abiotic stress conditions [150]. In *R. chinensis* cultivars, ‘Tineke’ showed greater abiotic stress tolerance, and ‘Hiogi’ abiotic stress tolerance was mediated by ABA-dependent signaling and glutathione metabolism. The cultivar ‘Tineke’ showed greater resilience through increased expression of *SnRK2*, *ABF*, *HSP*, *GSTs*, and *GSH1*, leading to improved photosynthetic capacity and water retention under salinity stress [151].

The Myo-inositol-1-phosphate synthase (MIPS) gene family, essential for membrane biosynthesis and signaling, also shows stress-responsive behavior in *R. chinensis*. *RcMIPS* downregulation in heat-sensitive lines correlated with reduced photochemical efficiency and disrupted carbohydrate metabolism, whereas drought-tolerant *Rosaceae* species exhibited MIPS upregulation accompanied by cis-regulatory elements such as ABRE, DRE, MYB, and MYC motifs [152]. Sugar transporters from the SWEET family have been associated with cold stress adaptation. In *R. beggeriana*, *SWEET2a* and *SWEET10c* were upregulated in leaves under low temperature, whereas their homologous genes, *RcSWEET2a* and *RcSWEET10c,* in *R. chinensis* ‘*Old Blush*’ exhibited cold sensitivity, suggesting genotype-specific sugar partitioning strategies under stress [153]. LBD TFs have been implicated in ABA signaling and abiotic stress regulation. In *R. rugosa*, salt-induced expression of *RrLBD39* (leaf expressed) and *RrLBD40* (root expressed) demonstrated their organ-specific roles in coordinating transcriptional and metabolic responses to salinity [94].

Proteins containing the BURP domain, which are associated with plant development and stress responses, have also been functionally validated in *R. chinensis*. *RcBURP4*, induced by salt, drought, and ABA treatments, improved drought tolerance when overexpressed in *Arabidopsis* via upregulation of *AtRD29A* and *AtRAB18*. Silencing *RcBURP4* reduced dehydration resilience [154]. SOD enzymes mitigate oxidative stress by scavenging ROS. In *R. hybrida*, *SOD2* expression correlated with reduced ion leakage and improved water status under drought, thereby enhancing stress tolerance [155]. Thaumatin-like proteins (TLPs), typically associated with pathogen defense, have recently been implicated in abiotic stress signaling. In *R. chinensis*, silencing *RcTLP6* increased salt sensitivity, whereas its interaction with *RcPR4*, *RcbHLH59*, and *RcMYB8* indicated a coordinated transcriptional and hormonal response under salinity stress [156]. The homeodomain leucine zipper (HD-Zip) TF family also plays an important role in osmotic adjustment. In *R. hybrida*, *RhHB1* regulates JA, (Ile) accumulation, and water homeostasis under dehydration. Silencing *RhHB1* reduced drought tolerance and disrupted osmotic balance [157]. The CBL–CIPK (calcineurin B-like proteins and CBL-interacting protein kinases) signaling network, crucial for calcium-mediated stress transduction, has been well characterized in *R. chinensis*. Multiple *RcCBL* and *RcCIPK* genes showed differential expression under heat, cold, drought, salt, and ABA treatments, highlighting their integrative role in calcium-dependent abiotic stress signaling [158].

## Cross-talk between TFs and hormone signaling in roses under stress adaptation

Recent studies in roses reveal complex TF and hormone regulatory modules that underpin abiotic stress adaptation through multilayered network integration. The *RcNAC091*–*RcWRKY71* cascade exemplifies this complexity. *RcNAC091* directly activates *RcWRKY71* and the ABA biosynthesis gene *RcNCED1*, forming positive feedback loops that coordinate ABA biosynthesis and signaling via *RcABF2* and *RcABI5* [79]. Similarly, *RcNAC72* functions as a central hub connecting ABA-dependent and ABA-independent pathways through *RcABF4*-mediated promoter activation and physical interaction with *RcDREB2A*, thereby linking ABA signaling with DREB/CBF-COR stress pathways, a pattern conserved in *Arabidopsis* [98]. WRKY TFs such as *RrWRKY1* integrate ABA synthesis (*RrNCED1*) and ABA-responsive TFs (*RrABF2*) with MAPK-mediated phosphorylation cascades [114], whereas *RhNAC31* bridges ABA and calcium signaling through simultaneous activation of ABA components (*ABI2, ABF4*) and Ca^+2^-responsive kinases (CIPK3) [100]. Cross-family integration also extends to zinc-finger proteins: *RrC_2_H_2_–8* responds rapidly to ABA as an early regulator of salt stress, paralleling the function of *Arabidopsis ZFP4/7* [89].

Calcium TF interactions further link hormone and stress signaling networks. Calmodulin (CaM3) activates WRKY and HSF members, with HSFA3–DREB2A modules integrating ABA-mediated osmotic stress responses [71]. Other master regulators such as *MBF1c* coordinate ET signaling through ERF pathways with HSF networks, whereas *NAC29/72* connect ABA signaling to heat-stress adaptation [123]. MYB family members, including *RmMYB108* and *RhMYB96*, modulate ET, JA, and ABA signaling by regulating ABA biosynthesis (*ABA3*) and signaling genes (*ABI1, ABI2, HAI1*) [129, 130]. Systems-level integration is further illustrated by *ERF109*, which governs hormone biosynthesis (YUC2) and multiple signaling pathways (ABA, JA, ET, and auxin), in coordination with *WRKY40*-mediated ABA sensitivity [131]. Similarly, *RcTINY2* links ABA biosynthesis and signaling to calcium and MAPK-dependent cascades [86].

Hormone TF feedback is evident in DREB regulation of gibberellin oxidases, connecting stress tolerance with GA homeostasis [137], and in bHLH-JA modules, where *MYC2* integrates jasmonate signaling with JAZ-mediated transcriptional feedback [144]. Mediator complex subunits also act as integration nodes. *RhMED15a* bridges ABA and JA signaling, coordinating hormone-dependent and independent stress-responsive genes through TF interactions [147, 148]. The ABA receptor SnRK2 ABF hierarchy further connects to DREB activation, reinforcing hormone TF transcriptional cascades during salt adaptation [151]. Additional cross-talk occurs through LBD TFs: *RrLBD25, RrLBD39,* and *RrLBD40* are each associated with distinct hormone pathways, auxin, JA, and ABA [94]. Moreover, the *RhHB1-RhLOX4* feedback circuit fine-tunes JA biosynthesis through transcriptional repression, optimizing stress response efficiency [157]. Collectively, these findings indicate that rose abiotic stress tolerance is governed by conserved, multilayered TF hormone signaling integration. This mechanistic framework extends beyond individual TF families, including interconnected regulatory networks that ensure coordinated responses to diverse environmental stresses.

## Post-translational and epigenetic regulation in roses under abiotic stress

Abiotic stresses impose major constraints on plant growth and productivity by triggering complex regulatory responses across multiple cellular levels. In *Rosa* species, two major systems, PTMs and epigenetic mechanisms, act both in parallel and synergistically to fine-tune stress adaptation. PTMs modify protein function after translation by adding chemical groups that influence protein stability, subcellular localization, and interaction dynamics, thereby enabling plants to mount rapid and coordinated defense responses [159, 160]. Key PTMs regulating stress responses include phosphorylation, acetylation, methylation, SUMOylation, ubiquitination, and glycosylation. These modifications form integral components of signal transduction cascades that reprogram metabolic and transcriptional networks during stress exposure. High-throughput proteomic analyses of PTM-regulated proteins have identified candidate regulators for engineering stress-resilient crops with improved productivity and environmental adaptability [161]. In parallel, epigenetic regulation mediates heritable changes in gene expression that occur without alterations in DNA sequence. Mechanisms such as DNA methylation, histone modification, chromatin remodeling, RNA-directed DNA methylation, and suppression of transposable elements are increasingly recognized as vital components of plant stress memory and long-term adaptation [162]. In *Rosa,* epigenetic modifications influence developmental plasticity and enhance resilience to fluctuating environments [163].

**Figure 6:**
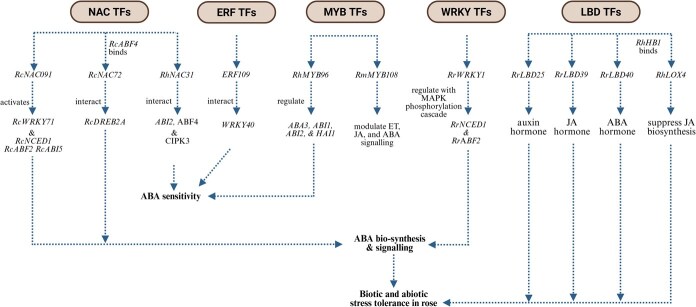
Integrated TF-hormone regulatory network governing stress adaptation in roses. The integrated regulatory network illustrates how diverse TF families coordinate hormone-mediated signaling to confer abiotic and biotic stress tolerance in roses. NAC TFs serve as upstream regulators, where *RcNAC091* activates *RcWRKY71* and ABA biosynthesis genes (*RcNCED1*, *RcABF2*, *RcABI5*), while *RcNAC72* interacts with *RcABF4* and *RcDREB2A* to link ABA-dependent and independent pathways. *RhNAC31* further bridges ABA and Ca^2+^ signaling by activating *ABI2*, *ABF4*, and *CIPK3*. ERF members, such as *ERF109*, interact with *WRKY40* to modulate ABA sensitivity and coordinate multiple hormone pathways (ABA, JA, ET, and auxin). MYB TFs (*RhMYB96*, *RmMYB108*) modulate ET, JA, and ABA signaling by regulating key biosynthetic and signaling genes (*ABA3*, *ABI1*, *ABI2*, *HAI1*), contributing to hormone balance and stress resilience. WRKY TFs (*RrWRKY1*) integrate MAPK phosphorylation cascades with ABA signaling via *RrNCED1* and *RrABF2*, reinforcing ABA biosynthesis and downstream responses. LBD TFs (*RrLBD25*, *RrLBD39*, *RrLBD40*) associate with auxin, JA, and ABA pathways, while *RhHB1–RhLOX4* modules suppress JA biosynthesis to fine-tune hormonal cross-regulation. Collectively, these interconnected TF–hormone circuits synchronize ABA biosynthesis, sensitivity, and signaling with other hormonal pathways, establishing a multi-layered transcriptional framework that enhances biotic and abiotic stress tolerance in rose plants.

The synergistic action of PTMs and epigenetic regulation equips roses with both short-term molecular flexibility and long-term genomic stability. PTMs allow immediate adjustments to protein function during stress exposure, whereas epigenetic modifications orchestrate durable transcriptional reprogramming. This dual-layered system confers physiological plasticity, enabling perennial ornamentals such as roses to maintain homeostasis and growth under prolonged or recurrent stress. Together, these mechanisms establish an integrated dynamic framework for stress adaptation in *Rosa*, offering promising prospects for molecular breeding and biotechnological innovation (Fig. 6).

## Phosphorylation

**Figure 7:**
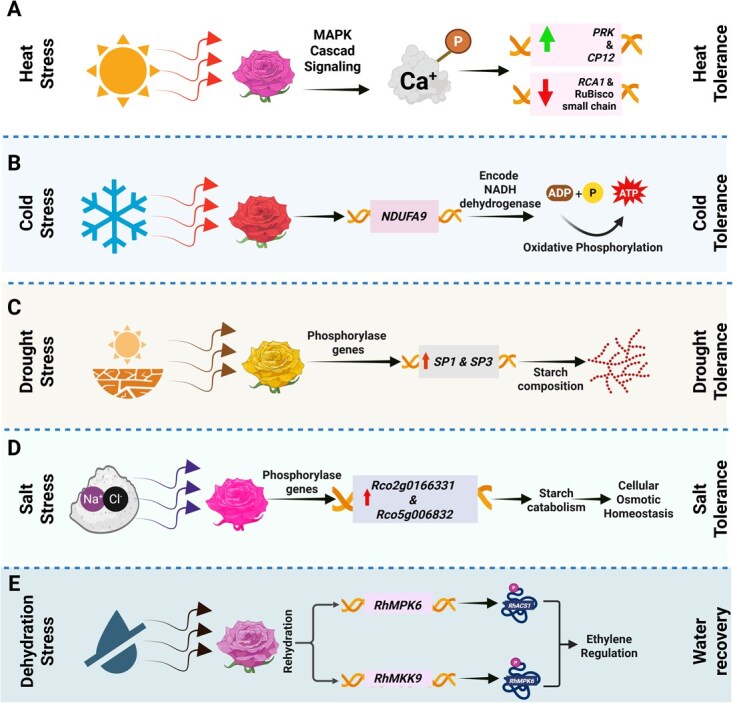
Phosphorylation-mediated regulatory mechanisms enhancing abiotic stress tolerance in rose plants. A. Heat stress activates the MAPK signaling cascade and causes phosphorylation of Ca^+^-dependent proteins which upregulate photosynthetic genes (*PRK* and *CP12*) and downregulate (RuBisco small chain and *RCA1*), which confers heat tolerance in *R. chinensis*. B. Cold stress activates the *NDUFA9* gene that encodes the NADH dehydrogenase subunit. It is involved in oxidative phosphorylation, which confers cold tolerance in *R. hybrida*. C. Drought stress upregulates the phosphorylase genes (*SP1* & *SP3*) that are involved in starch composition, which confers drought tolerance in *R. chinensis*. D. Salt stress upregulates the phosphorylase genes (*Rco2g0166331* & *Rco5g006832*) that are involved in starch catabolism and maintain cellular osmotic homeostasis, which confers salt stress in *R. chinensis*. E. Rehydration activates (*RhMPK6* & *RhMKK9*) genes that cause phosphorylation of *RhACS1* and *RhMPK6* proteins that regulate ET production, which confers water recovery in *R. hybrida*.

Phosphorylation is among the most prevalent PTMs in plants and plays a central role in signal transduction pathways that regulate responses to environmental stimuli [164]. In *Rosa* species, recent findings have elucidated phosphorylation-dependent mechanisms underlying adaptive responses to diverse abiotic stresses (Fig. 7). Under salt stress, multiple phosphorylation sites have been identified in regulatory proteins (Fig. 7D). In *R. rugosa, RrWRKY1* contains 64 serine (Ser), 20 threonine (Thr), and 7 tyrosine (Tyr) residues predicted to undergo phosphorylation, suggesting tight modulation of its activity through extensive phosphorylation [114].

In response to heat stress, *R. chinensis* activates rapid phosphorylation signaling through MAPK cascades, notably through *ANP1*, *YODA*, and calcium-dependent protein kinases such as *CPK13*, *CIPK2*, and *CIPK9 (*Fig. 7A*)*, within 0.5 to 2 hours after stress onset [123]. This signaling cascade regulates photosynthesis-related genes, with *PRK* and *CP12* upregulated, whereas the *RuBisCO* small chain and *RCA1* are downregulated, thereby modulating photosynthetic efficiency under high temperature. Cold stress adaptation also depends on phosphorylation-based pathways. In *R. hybrida*, *NDUFA9*, which encodes a subunit of NADH dehydrogenase participating in oxidative phosphorylation, exhibits a biphasic expression pattern under freezing conditions, showing initial suppression followed by later induction, indicating a function in late-phase cold acclimation [165].

During drought, *R. chinensis* displays differential expression of starch phosphorylase genes (Fig. 7C). *Rco4g0438671* (*SP1*) and *Rco5g0009141* (*SP3*) are markedly upregulated, likely contributing to osmotic adjustment through starch degradation. Conversely, *Rco2g0166331* and *Rco5g0068321* respond predominantly to salt stress by promoting carbohydrate catabolism but are repressed under heat stress [166]. Phosphorylation also regulates water-stress signaling in floral tissues (Fig. 7E). In *R. hybrida*, rehydration induces *RhMPK6*, which phosphorylates and stabilizes *RhACS1* in gynoecia, enhancing ET biosynthesis [167]. Upstream, *RhMKK9* phosphorylates *RhMPK6*, and its expression is epigenetically regulated through promoter demethylation during rehydration [168]. Additionally, phosphorylation of aquaporin *RhPIP2;1* at Ser273 facilitates nuclear accumulation of *RhPTM CEND*, suppressing growth while enhancing dehydration tolerance [169]. Another ABA-responsive protein, *RhRab5ip,* interacts with *RhPIP1;1* during flower opening, mediating vesicle trafficking between internal membranes and the plasma membrane under water-deficit conditions [170].

## Ubiquitination

In addition to phosphorylation, ubiquitination represents a major PTM that regulates stress responses in *Rosa* species. This reversible process entails the covalent attachment of ubiquitin molecules to specific lysine residues on target proteins, thereby altering their conformation, stability, and activity. Ubiquitination plays essential roles in transcriptional regulation, DNA repair, signal transduction, protein degradation, apoptosis, and translation [171]. The ubiquitination cascade is orchestrated by a hierarchical enzymatic system consisting of ubiquitin-activating (E1), ubiquitin-conjugating (E2), and ubiquitin ligase (E3) enzymes, together with deubiquitinating enzymes (DUBs) and the 26S proteasome complex, which collectively define substrate specificity and turnover rates [172].

Emerging evidence indicates that ubiquitination and related pathways participate actively in rose adaptation to abiotic stresses. Under cold stress, *R. hybrida* shows upregulated expression of genes encoding small ubiquitin-like modifiers (SUMO) and tubby-like proteins, both implicated in stress signaling and protein homeostasis [135]. During salinity exposure, *R. rugosa* cv. ‘Zizhi’ activates several ubiquitin-associated proteins, including ubiquitin C (*RRU03G01789.1*), a ubiquitin-like protein (*Rru03G018260.1*), and an E3 ubiquitin ligase (*Rru06G054150.1*), suggesting a critical role in maintaining proteostasis under ionic stress [93]. Heat stress has likewise been associated with ubiquitin-mediated proteolysis in roses. Transcriptomic profiling of self-grafted *R. chinensis* and heterografted *R. multiflora* lines revealed significant enrichment of genes related to the ubiquitin proteasome pathway under elevated temperatures [71]. Integrative transcriptomic and proteomic analyses in *R. damascena* also confirmed differential regulation of heat-responsive genes associated with ubiquitin-dependent protein turnover [6].

Specific ubiquitin genes show stable transcriptional patterns under water deficit, indicating their role in maintaining protein quality control during dehydration and rehydration. For instance, *RhUBI1*, *RhUBI2*, and *RhUBI6* showed consistent expression in *R. hybrida* petals, highlighting their suitability as reference markers in stress response studies [173]. Moreover, ubiquitin and ubiquitin-conjugating enzyme (UBC) genes have been validated as reliable reference genes for normalization of gene expression analyses under abiotic stresses such as heat, mechanical injury, and biotic challenges, as determined using stability ranking tools including BestKeeper, geNorm, and NormFinder [174].

## DNA methylation

**Figure 8:**
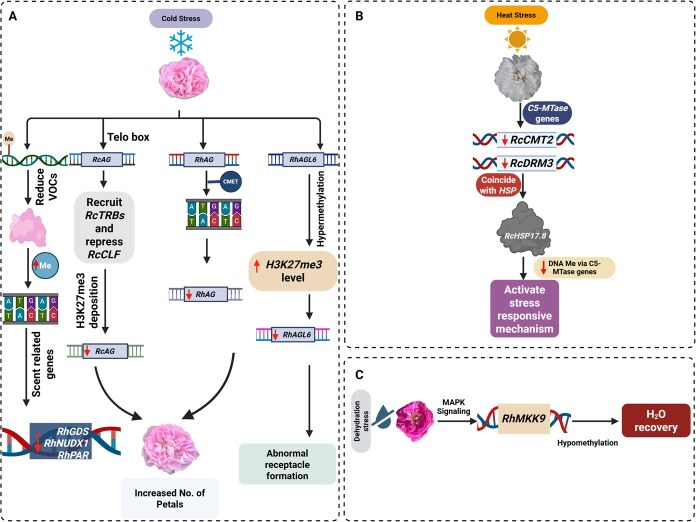
DNA methylation-mediated modulation under cold, heat, and water stress in rose plants. A. Cold stress induced the *RhAG* gene and enhanced DNA methylation of the *RhAG* promoter at CHH loci, leading to reduced *RhAG* expression and an increased number of petals in *R. hybrida*. In *R. chinensis*, cold stress expresses telo boxes in the second intron of the *RcAG* gene, which recruit *RcTRBs*, which form a repressor complex with *RcCLF*. This leads to low expression of *RcAG* through H3K27me3 deposition, resulting in an increased number of petals. In cold stress, higher temperature triggers *RhAGL6* hypermethylation and elevated H3K27me3 levels on its promoter, leading to downregulation of *RhAGL6* expression and abnormal receptacle formation in *R. hybrida*. In *R. chinensis*, chilling temperatures induced changes in DNA methylation, leading to a reduction in VOC emissions. This reduction increased DNA methylation at CHH loci, decreasing the expression of scent-related genes. B. Heat stress in *R. chinensis* significantly downregulated *C5-MTase* genes. This downregulation coincides with heat shock protein, which decreases DNA methylation through *C5-MTase* genes and activates the stress-responsive mechanism. C. In water stress, a key gene (*RhMKK9*) is induced in the MAPK signaling pathway during rehydration. These dynamic changes in DNA methylation, elevated methylation levels in the gene body, and hypomethylation in its promoter help in water recovery.

DNA methylation is a fundamental epigenetic modification that regulates transcription, maintains genome stability, and modulates chromatin architecture in both plant and animal systems [175]. In plants, methylation mainly occurs at cytosine residues in CG, CHG, and CHH contexts (where H represents A, T, or C) and serves to silence transposable elements, regulate gene expression, and preserve epigenomic integrity. Two primary forms, 5-methylcytosine (5mC) and N6-methyladenine (6 mA), are recognized for their regulatory functions under abiotic stress. These marks can form a type of ‘stress memory’ persisting through mitotic cell divisions and being reset once favorable conditions return. In some cases, such epigenetic imprints may be transmitted to progeny, contributing to transgenerational stress resilience [176]. In *R. hybrida*, DNA methylation modulates floral organ identity under cold stress (Fig. 8A). *Ma et al.* demonstrated that low temperature induces CHH hypermethylation of the *RhAG* promoter, suppressing *RhAG* expression and leading to stamen-to-petal conversion with increased petal number [177]. In *R. chinensis*, *Lu et al.* identified short interstitial telomeric motifs (telo boxes) within the second intron of *RcAG* that recruit *RcTRBs*, which assemble a repressive complex with *RcCLF* to deposit H3K27me3 marks, silencing *RcAG* under cold stress [178]. Similarly, *Jing et al.* reported that cold-induced suppression of *RhAGL6* correlates with promoter hypermethylation and elevated H3K27me3 levels, contributing to receptacle malformation in the ‘Peach Avalanche’ cultivar [179].

Heat stress exerts contrasting effects on methylation machinery. *Gangwar et al.* observed pronounced downregulation of C5-methyltransferase (C5-MTase) genes in *R. chinensis*, particularly *RcCMT2* and *RcDRM3*, with fold changes of 17.24 and 21.79, respectively (Fig. 8B). Ten C5-MTase genes in *R. chinensis* contribute to methylation-mediated stress regulation. Their suppression under heat stress coincided with increased transcription of *RcHsp17.8*, suggesting that reduced methylation enhances heat-responsive gene activation [180]. DNA methylation also modulates floral scent emission and water stress adaptation. *Xie et al.* reported that chilling treatment of ‘Crimson Glory’ roses suppressed VOC emission by increasing CHH methylation at the promoters of *RhGDS*, *RhNUDX1*, and *RhPAR*, thereby reducing transcript abundance of these scent-related genes [181]. During dehydration and rehydration cycles, *Chen et al.* found that the *RhMKK9*, *MAPKK* gene exhibits stress-induced expression changes regulated by methylation dynamics, characterized by hypermethylation within the gene body and hypomethylation at the promoter, which modulates ET biosynthesis and water recovery in *R. hybrida* floral tissues (Fig. 8C) [168].

## Molecular approaches to facilitate rose tolerance to biotic and abiotic stress

Molecular breeding has become a powerful strategy for mitigating the adverse effects of biotic and abiotic stresses in ornamental crops, particularly in *Rosa* spp. Its capacity to enhance stress resilience is increasingly recognized as essential for sustainable rose cultivation under changing climatic conditions. Several advanced molecular tools have been employed to accelerate the development of stress-tolerant rose genotypes. These include marker-assisted selection (MAS) [182], genome-wide association studies (GWAS), genome editing platforms such as CRISPR/Cas9 [183], and VIGS [78]. Complementary to these technologies, transcriptomic analyses provide critical insights into gene expression dynamics under stress, enabling the identification of candidate genes regulating key physiological and signaling pathways [184]. Understanding transcriptional and molecular responses under different stressors supports precision breeding for enhanced adaptability. Collectively, these molecular tools establish a robust framework for engineering roses with enhanced tolerance to drought, salinity, temperature extremes, and other environmental challenges while maintaining high ornamental quality and productivity.

## Mas

Molecular markers are defined as specific DNA sequences closely associated with genes of interest and inherited together with genomic regions governing important traits. With continuous advances in molecular marker technologies, breeders now possess highly effective tools for evaluating genetic diversity and accelerating the improvement of complex quantitative traits in *Rosa* spp., including those related to yield, floral quality, and stress resilience [185]. MAS enables the precise identification and selection of individuals carrying favorable alleles associated with abiotic stress tolerance. Among the available marker systems, single nucleotide polymorphisms (SNPs) and simple sequence repeats (SSRs) have become the most widely adopted in rose breeding programs because of their abundance, genome-wide distribution, and high resolution. Numerous SSR markers have been developed for genetic analysis and diversity studies in roses and related ornamental crops [186]. Although MAS itself does not generate novel genetic variation, it greatly improves breeding efficiency by enabling early and accurate selection of genotypes exhibiting desirable traits [187]. In *Rosa* spp., MAS has been successfully applied to identify genotypes with enhanced abiotic stress tolerance, such as drought and cold, which are primary constraints limiting global rose production. This approach supports the development of resilient cultivars capable of maintaining ornamental and physiological performance under suboptimal environmental conditions.

Recent advances in molecular marker development have further accelerated rose genetics and breeding by allowing dissection of complex traits. Early studies utilized restriction fragment length polymorphism (RFLPs), SSRs, and amplified fragment length polymorphism (AFLPs), whereas next-generation sequencing has enabled the identification of more than 68,000 SNPs, culminating in the development of the 68K WagRhSNP array. This array underpins high-density linkage mapping and GWAS [188, 189]. These resources have uncovered genomic regions associated with pigmentation, adventitious shoot regeneration, and stress responses, whereas quantitative trait locus (QTL) mapping has revealed loci for ornamental and stress-related traits [190–192]. The availability of complete diploid rose genome assemblies [4, 193] and diverse population resources now enables precise trait mapping, including for stress resistance. Transcriptome analyses have also identified candidate genes and pathways associated with adaptation to salt, cold, and heat stress, as well as defense against pathogens such as *B. cinerea*, *Podosphaera* spp. (powdery mildew), and *Peronospora sparsa* (downy mildew) [25, 123, 151, 194].

To date, most progress in developing molecular markers directly associated with stress tolerance has focused on biotic stresses. The *Rdr1* locus, which confers resistance to black spot disease, has been molecularly cloned and encodes a TIR-NB-LRR (Toll/interleukin-1 receptor-nucleotide-binding-leucine-rich repeat) protein. Tightly linked SNP and SSR markers now allow predictive use of *Rdr1* in breeding programs [195]. Comparative analyses revealed multiple expressed TNL copies at the *Rdr1* locus, providing valuable targets for cisgenic improvement. Similarly, *Rdr3* and *Rdr4* loci for black spot resistance have been mapped, although across broader genetic intervals, and marker refinement remains in progress [31, 196]. For powdery mildew, resistance is mediated by both major genes (e.g., *mlo*) and QTLs, with markers identified in *R. roxburghii* and related species [197]. The *Rpp1* locus conferring mildew resistance has also been fine-mapped and associated with specific diagnostic markers [198]. These developments collectively highlight the power of molecular markers as indispensable tools for dissecting stress resilience in roses. By linking genotype to phenotype through SNP arrays, QTL mapping, and stress-linked loci, breeders can efficiently accelerate the development of cultivars exhibiting durable resistance to both abiotic and biotic stresses.

## GWAS in roses

GWAS has become a powerful strategy for identifying genetic variants associated with complex traits by analyzing millions of SNPs across the genome. This high-resolution approach leverages natural genetic variation within diverse populations, making it particularly suitable for dissecting polygenic traits influenced by multiple loci. With advances in genomics and high-throughput DNA microarray technologies, GWAS enables simultaneous exploration of numerous phenotypic traits on the genome-wide scale. In *Rosa* spp., GWAS has been instrumental in identifying candidate genes and genomic regions related to pigment biosynthesis, including anthocyanin and carotenoid pathways, as well as adventitious shoot formation [5]. These discoveries provide valuable insights into the genetic architecture of physiological and economically important traits. Importantly, GWAS holds strong potential for uncovering alleles associated with abiotic stress tolerance, including drought resistance, salinity adaptation, and temperature resilience, which are governed by complex regulatory networks. By elucidating the genetic basis of these traits, GWAS supports precision breeding strategies and enables the integration of marker-assisted and genomic selection approaches to accelerate the development of stress-resilient rose cultivars.

## Genome editing and gene silencing technologies

Genome editing has transformed functional genomics and modern plant breeding by allowing precise and predictable modifications at specific genomic loci. This technology employs sequence-specific nucleases (SSNs) to induce double-strand breaks (DSBs) in DNA, which are subsequently repaired via nonhomologous end joining (NHEJ) or homology-directed repair (HDR) pathways. These mechanisms facilitate targeted insertions, deletions, or replacements, enabling functional alteration of genes associated with agronomic or stress-related traits [199].

Among available genome editing systems, the Clustered Regularly Interspaced Short Palindromic Repeats/CRISPR-associated protein 9 (CRISPR/Cas9) platform has gained widespread use because of its simplicity, high efficiency, and programmability. CRISPR/Cas9 allows targeted gene disruption regulation, or replacement, and has been widely applied to enhance abiotic stress tolerance in various crop species.

## VIGS

VIGS is a rapid and efficient reverse genetics tool for functional genomics in plants, offering substantial potential for molecular breeding in ornamentals, including *Rosa* spp. [200]. VIGS utilizes viral vectors to transiently suppress the expression of target genes, enabling the functional characterization of candidate genes without requiring stable transformation. This system has been used to investigate plant development, stress physiology, pathogen defense, and secondary metabolism. In rose research, VIGS represents a cost-effective platform for elucidating gene function and identifying targets associated with abiotic stress tolerance, floral coloration, and disease resistance [201]. It is particularly advantageous for studying complex regulatory networks and validating genes prior to genome editing. Recent work has demonstrated that VIGS-mediated silencing of negative regulators enhances tolerance to abiotic stresses such as drought and salinity [80]. For instance, suppression of genes participating in ET biosynthesis or signaling has been proposed to reduce stress-induced senescence and maintain physiological stability under adverse conditions. As a transient gene silencing method, VIGS accelerates gene function validation and supports precision breeding in ornamental plants where stable transformation remains technically challenging.

## CRISPR/Cas9

The CRISPR/Cas9 system has rapidly emerged as a transformative genome-editing platform, providing high precision and efficiency for trait improvement in ornamental plants, including *Rosa* spp. [202]. This RNA-guided endonuclease system has been widely implemented in both basic plant research and applied breeding, enabling site-specific modification of endogenous genes with remarkable accuracy. CRISPR/Cas9 has been used to manipulate floral organ identity, pigmentation pathways, flowering time, volatile biosynthesis, and stress tolerance traits [203]. Traditional breeding in roses is often limited by long generation cycles, genomic complexity, and high heterozygosity. CRISPR/Cas9 circumvents these challenges by facilitating direct modification of key genes underlying desirable phenotypes. Editing genes related to osmotic regulation, ROS detoxification, and hormonal signaling can substantially improve tolerance to drought, salinity, and temperature extremes. With the availability of high-quality rose genome assemblies and comprehensive functional annotations, CRISPR/Cas9 now enables detailed dissection of stress-responsive pathways and accelerates translation of genomic insights into improved cultivars [204]. This precision breeding tool not only expedites the creation of climate-resilient rose varieties but also deepens understanding of the molecular frameworks that govern stress adaptation in perennial ornamental plants.

## Future perspectives

Substantial progress has been achieved in elucidating the molecular mechanisms underlying biotic and abiotic stress adaptation in roses; however, a critical gap remains in the functional validation of candidate genes identified through transcriptomic and genomic studies. To translate these discoveries into practical breeding outcomes, an integrated framework linking gene discovery, molecular marker application, and precision genome editing is required.

The proposed framework begins with GWAS and QTL mapping to identify genomic regions statistically associated with stress tolerance traits across diverse rose germplasm. Candidate genes within these regions can then be validated through functional genomics approaches, including CRISPR/Cas9-mediated knockout and comparative transcriptomic profiling between contrasting phenotypes, to confirm their roles in stress-responsive pathways. Once validated, these genes provide the foundation for marker development, enabling MAS to improve parent selection efficiency and accelerate breeding cycles. This workflow is particularly valuable for complex quantitative traits related to abiotic stress tolerance that are difficult to evaluate during early developmental stages. For resistance traits, MAS supports marker-assisted backcrossing and gene pyramiding, allowing breeders to combine multiple favorable alleles while minimizing linkage drag through dense marker coverage of target regions. Finally, CRISPR-based precision editing can be employed to introduce advantageous alleles or modify regulatory sequences directly within elite cultivars, enabling targeted improvement even in polyploid genetic backgrounds.

This cyclical integration, from GWAS-based discovery through functional validation to MAS implementation and precision genome editing, effectively bridges the gap between genomic research and applied rose breeding. When combined with multi-omics data integration, including transcriptomic and metabolomic profiling together with high-resolution phenotyping, this comprehensive strategy provides a clear roadmap for developing climate-resilient rose cultivars with durable stress tolerance and sustained ornamental quality under the growing challenges of global climate change.

## Conclusion

Biotic and abiotic stresses remain major constraints to rose cultivation, undermining both the ornamental appeal and commercial value of this economically significant crop. The capacity of roses to withstand environmental challenges and pathogen attacks is governed by complex molecular networks, including transcriptional regulation, PTMs, and epigenetic mechanisms. Recent progress has revealed key regulatory genes and signaling pathways that mediate rose responses to drought, salinity, temperature extremes, and major pathogens such as black spot, powdery mildew, and gray mold. These advances provide promising molecular targets for developing stress-resilient cultivars. This review consolidates current knowledge of the molecular mechanisms underlying biotic and abiotic stress responses in roses, emphasizing regulatory networks that may differ from those characterized in model plants. Whereas earlier studies largely addressed individual stress factors or model species, recent findings demonstrate that roses coordinate responses to multiple stressors through integrated TF networks, hormone signaling cascades, and epigenetic modifications. Comparative analyses of biotic and abiotic stress mechanisms highlight both convergent and divergent molecular strategies, establishing a framework for understanding stress adaptation in perennial ornamental crops. Current molecular approaches have successfully identified stress-responsive gene networks through transcriptomic and genomic analyses and have provided precision breeding tools via GWAS and genome editing. Nonetheless, several limitations persist, including the insufficient functional validation of candidate genes, low transformation efficiency, inadequate field validation, and lack of standardized stress-screening protocols. Future research should prioritize the functional validation of key genes using CRISPR/Cas9 and VIGS technologies, the development of efficient transformation systems for *Rosa* spp., and field-based evaluations to bridge the gap between laboratory discovery and practical application. Integrating molecular insights into breeding pipelines will be essential for developing rose cultivars that combine high aesthetic value with enhanced tolerance to both biotic and abiotic stresses. Given the accelerating effects of climate change on horticultural systems, elucidating the molecular and regulatory bases of stress adaptation in roses is critical for ensuring the long-term sustainability, productivity, and global competitiveness of rose cultivation.
